# Patient‐Reported Outcome Measures in Fetal Medicine: A Pilot Feasibility Study

**DOI:** 10.1002/pd.70013

**Published:** 2025-11-04

**Authors:** N. M. T. H. Crombag, B. Teeuwen, E. M. P. Akkerman, B. M. E. Adriaanse, A. L. Depla, A. Franx, A. J. M. Oerlemans, D. Stemkens, L. Henneman, M. N. Bekker

**Affiliations:** ^1^ Department of Obstetrics and Gynaecology Wilhelmina Children's Hospital Utrecht Utrecht University Utrecht the Netherlands; ^2^ Department of Obstetics and Gynaecology Erasmus Medical Centre Rotterdam the Netherlands; ^3^ IQ Health Science Department Radboud University Medical Center Nijmegen the Netherlands; ^4^ VSOP‐Patient Alliance for Rare and Genetic Diseases Utrecht the Netherlands; ^5^ Department of Human Genetics Amsterdam UMC Location Vrije Universiteit Amsterdam the Netherlands

## Abstract

**Objective:**

A core set of generic Patient Reported Outcome Measures (PROMs) was recently developed to collect information from patients about their health status and quality of life. This study aims to: (1) identify relevant Patient Reported Outcome Measures (PROMs) from this core set for parents facing a fetal anomaly diagnosis and determine their optimal use and (2) assess the usability and feasibility of the adapted setting‐specific PROMs in a Dutch Fetal Medicine Department.

**Method:**

A diverse expert panel of parents and healthcare professionals selected relevant PROMs along with their optimal timing and application. In a subsequent pilot feasibility study, parents completed the PROMs and discussed results with professionals. Responses were converted to T‐scores using the PROMIS short forms. Usability and feasibility were assessed via questionnaires.

**Results:**

Twenty‐eight participants (19 parents, 9 professionals) agreed on two key PROMs: “ability to participate in social roles” and ‘emotional distress (anxiety and depression)’. In the pilot study (*n* = 32; 21 parents, 11 professionals), PROMs were completed in 5.9 min on average, with participants opting to complete PROMs digitally from home. Parents found PROMs useful for enhancing communication with their partners and healthcare providers. The study identified the need for case managers, training on interpreting PROM results, a user‐friendly Information Technology (IT) platform, and customized PROMs.

**Conclusion:**

This study shows potential benefits of PROMs in Fetal Medicine but encountered challenges regarding complexity, professional engagement, and time constraints in practice.

## Summary

- What is already known about this topic?
  ◦ The suspicion of a fetal anomaly during pregnancy induces significant psychosocial stress for prospective parents and increases the need for tailored, personal, psychosocial prenatal care;
  ◦ The use of patient‐reported outcome measures (PROMS) in Fetal Medicine is limited;
  ◦ A core set of generic PROMs has shown to support patient‐centered care and increase overall quality of care.
- What does this study add?
  ◦ The ‘ability to participate in social roles' and ‘emotional distress (anxiety and depression)’ are key PROMs for parents facing (suspected) fetal anomalies;
  ◦ Implementing PROMs in Fetal Medicine requires continuity of care, professional training, and integrated Information Technology tools;
  ◦ The unique context of Fetal Medicine necessitates the development and validation of PROMs set specifically tailored to parental needs.

## 1 Introduction

Prenatal screening is increasingly conducted by non‐invasive prenatal testing (NIPT) for fetal chromosomal disorders, as well as the first‐trimester anomaly scan (FTAS) and second‐trimester anomaly scan (STAS) for fetal structural anomalies. In the last decades, the accuracy of these prenatal tests has significantly improved, and the scope has further expanded. Moreover, the uptake of prenatal screening has increased throughout recent years [1, 2].

Despite advancements in prenatal testing, parents faced with a suspected diagnosis of a fetal anomaly in their unborn child encounter significant dilemmas. The care journey involves many uncertainties which cause distress for prospective parents [3, 4, 5]. Antenatal decisions—such as pursuing confirmatory diagnostic testing after a positive screening result, deciding whether to continue or terminate a pregnancy, preparing for the birth of a child with a congenital anomaly, or considering fetal treatment—carry substantial consequences [3, 4, 5, 6, 7]. These decisions are personal and challenging, necessitating personalized counseling and support [3, 4, 8].

Supporting future parents facing a (suspected) fetal anomaly requires a thorough understanding of their unique needs. Patient‐reported outcome measures (PROMs) capture outcomes directly from the patient, including health, quality of life, symptoms, and functional status related to disease or condition. These outcomes were assessed through validated questionnaires. Collecting and using PROMs improves patient‐clinician communication, identifies unnoticed symptoms, and may improve health outcomes [3, 4, 8]. PROMs focus on patients' well‐being and enhance healthcare quality [9, 10].

A core set of seven *generic PROMs* was recently developed by the Program on Outcome‐Based Care in the Netherlands [3, 4, 8]. This set is designed for broad application across various medical fields and includes measures of “quality of life,” “overall health perception,” “physical functioning,” “ability to participate in social roles,” “emotional distress (anxiety and depression),” “fatigue,” and “pain intensity” [11, 12]. Although PROMs are intended to enhance the quality of care, they are not yet available for decision support among prospective parents confronting fetal anomalies during pregnancy [3, 4, 8].

Hence, the objectives of this study are twofold: (1) To identify relevant PROMs for parents facing a fetal anomaly diagnosis and determine their optimal use and (2) to explore the usability and feasibility of these adapted setting‐specific PROMs in a Dutch Fetal Medicine Department.

## 2 Methods

### 2.1 Setting

This study was carried out from January 2023 to September 2023 at the Fetal Medicine department within the University Medical Center Utrecht (UMCU) in the Netherlands. The Dutch perinatal care system has a two‐tiered model: community midwives handle low‐risk patients, and obstetricians manage medium‐ and high‐risk patients in hospital. Community midwives and medium‐risk obstetricians refer patients to tertiary hospitals with a Fetal Medicine department if suspicion of fetal anomalies arises from prenatal screening. The UMCU is a tertiary Fetal Medicine referral center serving the central region of the Netherlands and beyond.

**FIGURE 1:**
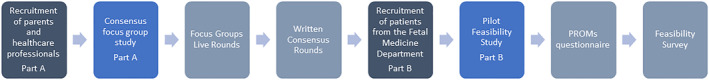
Study design and subsequent phases.

To achieve our objectives, this study consisted of two parts: (a) a consensus focus group study and, (b) a pilot feasibility study. Figure 1 provides a visual overview of the study design.

- Consensus focus group study

### 2.2 Study Population

Parents were recruited through the Dutch Patient Alliance for Rare and Genetic Diseases (VSOP), which alerted potential participants via organizations such as the Dutch Down Syndrome Foundation, Platform Congenital Diaphragmatic Hernia, Patient Alliance Congenital Heart Disease, Dutch Spina Bifida Foundation, and Knowledge Center Palliative Care, using their social media platforms. Potential participants could express their interest by sending an email to the researcher (NC). Additionally, patients were recruited at the Fetal Medicine department of UMCU.

Parents were eligible to participate if they had experienced a pregnancy diagnosed with a fetal anomaly between 2015 and 2022, regardless of whether they chose to continue or terminate the pregnancy. There were no restrictions based on gestational age or the timing of the diagnosis. Parents were excluded if they were under 18 years of age, had limited fluency in Dutch, or had a multiple pregnancy (twin, triplet).

Healthcare professionals were selected based on their expertise in caring for parents facing a fetal anomaly diagnosis, such as midwives, gynecologists, clinical geneticists, pediatric specialists, neonatologists, sonographers, and social workers. All healthcare professionals were based in the region where the UMCU Fetal Medicine Department is located.

### 2.3 Study Design

**FIGURE 2:**
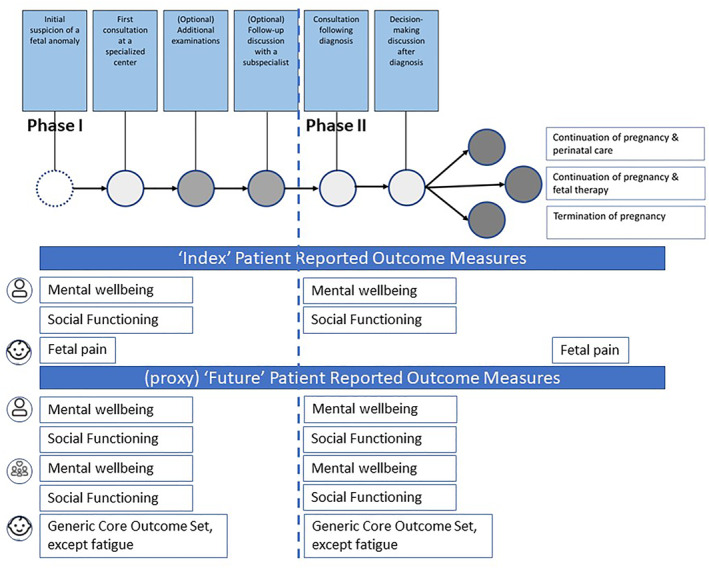
Overview of Patient Reported Outcome Measures (PROMs) selected from the core set of generic PROMs mapped on the Fetal Medicine care pathway. *Phase I: Timeline from suspected to confirmed diagnosis; Phase II: Timeline from confirmed diagnosis to management of pregnancy (continuation or termination).*

This qualitative focus group study aimed to reach consensus among stakeholders on the selection, timing, and use of a core set of seven generic PROMs tailored to Fetal Medicine, referred to as *adapted setting‐specific PROMs*. The focus group discussions involved three distinct groups: parents who continued their pregnancy after a fetal anomaly diagnosis, parents who terminated their pregnancy following a fetal anomaly diagnosis, and healthcare professionals involved in the care of such parents. Using a conversation guide (Supplementary I, conversation guide), each group was asked to identify: (a) relevant PROMs for the Fetal Medicine care pathway, (b) the appropriate timing for discussing PROMs with parents, (c) how to use it , and (d) the appropriate person to discuss PROM results with parents. Researchers M.B. and N.C. developed a care pathway template, which was subsequently validated by the research project group. The care pathway template includes six contact points with healthcare professionals: (1) Initial suspicion of a fetal anomaly; (2) First consultation at a specialized center; (3) (Optional) Additional examinations; (4) (Optional) Follow‐up discussion with a subspecialist; (5) Consultation following diagnosis; and (6) Decision‐making discussion after diagnosis. This care pathway template served as a framework for mapping relevant PROMs during the focus groups (Figure 2).

A researcher (N.C.) moderated the focus groups, with junior doctors (S.K., D.V., A.D.) taking field notes. The focus groups took place at the UMCU in January and February 2023. The sessions lasted between 63 and 125 min and continued until narrative consensus on the PROM set was reached. Initially, consensus was reached if participants agreed with the meeting notes and conclusions derived from the sessions. Audio recordings were made during consensus rounds and deleted after the meeting notes were finalized. While the focus groups were not transcribed, detailed meeting notes were taken for each group. These notes captured the consensus on how the relevant PROMs should be used, at what point in time, and by whom they should be discussed. The Miro digital whiteboard facilitated the process during both meetings, with screenshots included in the meeting notes (DPX%20‐%20Zorgpad%20PROMs%20foetale%20geneeskunde%20‐%20Miro). An overview of the consensus on PROMs was shared with participants after the sessions for feedback and correction, and finalized following the incorporation of minor suggestions. At this stage, it was considered a consensus across the three focus groups The final product was referred to as *‘adapted setting‐specific PROMs'*. To enhance the rigor of the study, the 32‐item Consolidated Criteria for Reporting Qualitative research checklistwas used (COREQ) (Supplementary II, COREQ).

b. Pilot feasibility study

### 2.4 Study Population

The pilot study included both parents and healthcare professionals. Parents were recruited at the Fetal Medicine department of the UMCU between May 2023 and September 2023. Parents were eligible if the index pregnancy was suspected or diagnosed with a fetal anomaly. There were no gestational age or diagnosis timing restrictions, except for a prior prenatal test indicating a fetal anomaly. Exclusion criteria for parents were being under 18 years of age, limited Dutch fluency, and multiple pregnancies.

Healthcare professionals included specialists from the UMCU Fetal Medicine Department and their collaborating partners across the wider Utrecht region and those who discussed the *adapted setting‐specific PROMs* with one of their patients.

### 2.5 Study Design

Within a 4‐month pilot (May 2023–September 2023), a cross‐sectional feasibility study with parents and professionals was conducted to evaluate the feasibility and usability of the *adapted setting‐specific PROMs* in the Fetal Medicine context.

### 2.6 Data‐Collection

Following informed consent, parents received PROM questionnaires via email, which they completed prior to their next consultation. The results were collected using Castor [13], converted into relevant scores, and subsequently discussed during the consultations. Afterward, participants completed the feasibility survey to provide feedback on the usability and feasibility of the *adapted setting specific PROMs*.

Before the pilot, healthcare professionals attended a presentation on the use of the *adapted setting specific PROMs* and received brief oral instructions before patient visits. During the pilot, they assessed PROM questionnaires during routine consultations and completed a feasibility survey at the end of the study regarding PROM assessment in routine visits.

### 2.7 Adapted Setting‐Specific PROMs

The *adapted setting‐specific PROMs* included 12 questions addressing the two PROMs identified in the consensus focus group study: “emotional distress (anxiety and depression)” and “ability to participate in social roles”. The sections consisted of four statements with five multiple‐choice answer options, ranging from “never” to “always.” In this study, we employed the Dutch‐Flemish PROMIS v1.0 4a short forms [11, 12]. PROMIS uses a T‐score metric in which 50 is the mean of a relevant reference population and 10 is the standard deviation (SD) of that population. Raw participant scores and converted T‐scores were calculated and analyzed using the pre‐established reference from the PROMIS short forms [11, 12]. Reference T‐score ratings by severity were as follows: T‐score for “emotional distress (anxiety and depression)”: Normal < 55, Mild: 55–60, Moderate: 60–70, Severe: > 70. T‐score for “Ability to participate in social roles”: Normal > 46, Mild 41–46, Moderate: 32–41, Severe: < 32. A full version of the questionnaire can be found in Supplementary III, the *adapted setting specific PROMs questionnaire*.

### 2.8 Feasibility Survey and Analysis

To assess feasibility, a separate feasibility survey was created for parents and healthcare professionals based on a previous multidisciplinary focus group study, and adapted for this study [14]. The questionnaires included multiple‐choice questions with opportunities for open‐ended answers. Data from the feasibility survey were recorded in Castor [13] and analyzed using R‐studio [15] employing descriptive statistics of the quantitative data. Qualitative data from the free text answers were thematically analyzed to enrich the survey findings, providing tentative insights from participants' responses.

### 2.9 Ethical Approval

The Medical Ethical Committee of the UMCU reviewed the protocol (no. 22U‐0426). The Committee declared that the Medical Research Involving Human Subject Act (WMO) did not apply to the present study and therefore exempted the protocol from needing further approval.

## 3 Results

- Consensus focus group study

Three distinct focus groups were conducted with 28 participants: (a) parents who had a history of a fetal anomaly and continued their pregnancy after a prenatal diagnosis of a fetal anomaly (*n* = 9), (b) parents who had a history of a fetal anomaly and opted for termination (*n* = 10), and c) healthcare professionals involved in care of parents receiving a diagnosis of a fetal anomaly (*n* = 9). Participants' characteristics are shown in Table 1.

**TABLE 1:** Participant characteristics and focus group (*n* = 3) structure.

Focus groups	Type of profession/fetal anomaly	Number, *n*
Healthcare professionals (*n* = 9)	Neonatologist	2
	Pediatric cardiologist	1
	Geneticist	1
	Social worker	1
	Community midwife	1
	Sonographer	2
	Fetal gynecologist	1
Parents who received a prenatal diagnosis of a fetal anomaly and choose to continue the pregnancy (*n* = 9)	Trisomy 21	6 (mothers)
	Congenital heart defect	2 (mothers)
	Spina bifida	1 (mothers)
Parents who received a prenatal diagnosis of a fetal anomaly and chose to terminate the pregnancy (*n* = 10)	Trisomy 21	2 (mother and father)^a^
	Congenital heart defect	2 (mother and father)^a^ ^,^ ^b^
	Spina bifida	1 (mother)
	Neurological anomaly	1 (mother)
	Trisomy 18	1 (father)
	Rare genetic condition	2 (mother and grandmother)^a^
	Hydrocephalus	1 (mother)
	Total	28

^a^ Belonging to the same family.

^b^ Terminated two pregnancies due to congenital heart defects, one of which also had trisomy 21.

Initially, both parents and professionals worried that using questionnaires for parents expecting a baby with a fetal anomaly might diminish the human aspect of care. However, after discussion, participants agreed that PROMs could facilitate deeper conversations about parental mental health during consultations.

All three focus groups agreed that Fetal Medicine addresses two main types of outcomes: (1) those related to the health and functioning of both the parents and the fetus during the index pregnancy, and (2) those concerning the future well‐being of the child and family, which are crucial for decision‐making about whether to continue the pregnancy. It was agreed that PROMs should focus on outcomes that are relevant to parents, families (including both parents and siblings), and the fetus/future child. PROMs related to the current pregnancy were referred to as “Index PROMs”, while those used for decision‐making about the future concerning the child were labeled “Future PROMs”. If the PROMs pertained to the fetus or future child, they were designated as “Proxy Future PROMs”.

During the focus group sessions, participants arranged the generic PROM core outcome set chronologically based on the care pathway, assigning PROMs to parents, families (parents and siblings), or the fetus/future child. Consensus was reached on the importance of “ability to participate in social roles” and “emotional distress (anxiety and depression)” as key “Index PROMs” during pregnancy, further referred to as *adapted setting‐specific PROMs*. The other PROMs were deemed irrelevant as parents indicated that factors such as “quality of life,” “overall health perception,” “physical functioning,” “fatigue,” and “pain intensity” were not their primary concerns during the challenging and uncertain period following their fetus's diagnosis. Parents also identified “fetal pain” as a relevant proxy outcome. “Future PROMs” for parents and families reflected the “Index PROMs”, while all proxy PROMs for the future child, except fatigue, were considered relevant.

Participants agreed that the Fetal Medicine care pathway could be divided into two phases: Phase I, from suspected to confirmed diagnosis, and Phase II, from confirmed diagnosis to pregnancy management (continuation or termination), with identified PROMs deemed essential for both phases (Figure 2).

All groups recommended adding “parental stress” to the core set, highlighting the importance of assessing stress beyond anxiety and depression. Healthcare professionals preferred maternal fetal specialists to discuss PROMs with parents, while parents preferred Fetal Medicine case managers (specialized professionals in Fetal Medicine) for “Index PROMs” and pediatric specialists for “Future PROMs”.

After the first round of each focus group, substantial consensus was reached. The second and final round involved written feedback from all participants on the draft care pathway and PROM arrangement.

b. Pilot feasibility study

For the pilot feasibility study, the “Index PROMs” selected from the focus groups—“ability to participate in social roles” and “emotional distress (anxiety and depression)”—were used, focusing on parents with a pregnancy involving a suspected or detected fetal anomaly, referred to as the *adapted setting‐specific PROMs*.

Of the 32 parents approached for the pilot feasibility study, 27 participated. Reasons for non‐participation included emotional burden, disinterest, or unspecified reasons. Supplementary IV presents prenatal diagnoses and pregnancy outcomes. Participants had a mean age of 30 years (range 22–36). Eleven obstetric healthcare professionals discussed PROMs with prospective parents and evaluated their feasibility in clinical practice. The group included obstetricians (*n* = 3), community midwives (*n* = 3), Fetal Medicine doctors (*n* = 3), and obstetric social workers (*n* = 2).

### 3.1 Adapted Setting‐Specific PROM Scores of Parents

Table 2 represents the T‐scores for “emotional distress (anxiety and depression)”, and “ability to participate in social roles”. Most parents showed a mild to moderate increase in anxiety levels, with normal scores for “depression” (T‐score < 55) and “ability to participate in social roles” (T‐score > 46)) remaining within the normal range, based on reference norms [11, 12]. To assess whether the observed T‐scores differed meaningfully from the general population mean (*T* = 50), exploratory one‐sample t‐tests were conducted. A subgroup analysis was performed to compare parental T‐scores across two phases: Phase I (from suspected to confirmed diagnosis; *n* = 7) and Phase II (from confirmed diagnosis to pregnancy management; *n* = 20). Phase II participants showed higher mean anxiety scores (μ = 60.2, 95% CI [56.6, 63.7]) and mildly elevated depression scores (μ = 52.4, 95% CI [47.5, 57.3]) compared to Phase I participants (anxiety: μ = 57.0, 95% CI [52.73, 61.23]; depression: μ = 47.4, 95% CI [42.86, 51.99]).

**TABLE 2:** T‐scores of PROMIS emotional distress (anxiety and depression)' and “ability to participate in social roles”.

	All participants (*n* = 27)	Phase I (*n* = 7)	Phase II (*n* = 20)
Anxiety	μ = 58.6	μ = 57	μ = 60.2
*T*‐score	95% CI [56.1, 61.4]	95% CI [52.73, 61.23]	95% CI [56.6, 63.7]
Depression	μ = 50.2	μ = 47.4	μ = 52.4
*T*‐score	95% CI [46.9, 53.5]	95% CI [42.86, 51.99]	95% CI [47.5, 57.3]
Ability to participate in social roles	μ = 51.9	μ = 52.1	μ = 51.9
*T*‐score	95% CI [48.2, 55.7]	95% CI [45.2, 58.9]	95% CI [47.1, 56.6]

*Note:* The mean (μ) and 95% confidence interval of the total *T*‐scores of “emotional distress (anxiety and depression)” and “ability to participate in social roles” by subgroup of Phase I and Phase II (Phase I: Timeline from suspected to confirmed diagnosis; Phase II: Timeline from confirmed diagnosis to management of pregnancy (continuation or termination)). Reference *T*‐score ratings ^11^: *T*‐score for Anxiety and Depression: Normal < 55, Mild: 55–60, Moderate: 60–70, Severe: > 70. *T*‐score for “Ability to participate in social roles”: Normal > 46, Mild 41–46, Moderate: 32–41, Severe: < 32.

### 3.2 Parental Perspectives on the Use and Feasibility of Adapted Setting‐Specific PROMs

Of the 27 parents who initially participated in the pilot, 78% (*n* = 21) completed the feasibility survey. Three participants withdrew for reasons including pregnancy termination, disinterest, or unwillingness, while three others left without specifying reasons.

Participants reported an average completion time of 5.9 min for the *adapted setting‐specific PROMs* (range 2–15 min) (Table 3). Most participants found the time required for completing the PROMs acceptable, with 81% rating it as adequate and 19% as short. None of the participants considered it too long. Participants preferred completing the PROMs on a smart phone device, via a website, and from home. While 81% needed no assistance in completing the PROMs, those who did consulted their partners. Open‐ended answers suggested adding ‘open text areas' for clarifying and motivating multiple‐choice answers and emphasized the need for a clear time frame for reflection. Most Participants (62%) also found discussing the PROMs with their partners a positive experience.

**TABLE 3:** Parent perspectives regarding the use of PROMs in Fetal Medicine, total *N* = 21.

Time to complete (minutes), mean (min‐max)				
5.9 (2–15)				
Acceptability of time spent, *n* (%)				
Too long	0 (0)			
Long	0 (0)			
Good	17 (81)			
Short	4 (19)			
Too short	0 (0)			
Preferred device, *n* (%)^a^				
Phone/Tablet (application)	2 (10)			
Phone/Tablet (website)	15 (71)			
Computer (website)	1 (5)			
On paper	3 (14)			
Other	1 (5)			
Preferred location, *n* (%)				
At home	15 (71)			
In the waiting room	4 (19)			
Other	2 (10)			
Assistance needed when answering, *n* (%)				
No, alone	17 (81)			
Yes, partner	4 (19)			
Yes, other	0 (0)			
Discuss PROM answers with healthcare professional, *n* (%)				
All answers	2 (10)			
Only deviant score answers	7 (33)			
Anonymous use	12 (57)			
Don't use PROMS at all	0 (0)			
Preferred care professional to discuss PROMs with *n* (%)^a^				
Perinatologist	7 (27)			
Clinical midwife	7 (27)			
Fetal medicine doctor	4 (15)			
Specialized obstetric nurse	2 (8)			
Medical social worker	2 (8)			
Clinical geneticist	1 (4)			
Neonatologist	0 (0)			
Specific medical specialist	0 (0)			
Other	3 (11)			
Transfer answers to new professional, *n* (%)				
Yes	14 (67)			
No	7 (33)			

**Statements on completing PROMs, *n* (%)**	**Agree**	**Neutral**	**Disagree**	**Not Applicable**
Goal clear	17 (81)	4 (19)	0 (0)	0 (0)
Explanation understood	17 (81)	4 (19)	0 (0)	0 (0)
Flyer clear	8 (38)	5 (24)	0 (0)	8 (38)
Understood the questions well	18 (86)	2 (10)	1 (5)	0 (0)
Capable to fill in all questions	18 (86)	1 (5)	2 (10)	0 (0)
Pleasant to discuss with partner	13 (62)	8 (38)	0 (0)	0 (0)
Pleasant to discuss with professional	8 (38)	4 (19)	2 (10)	7 (33)
Supports consultation preparation	7 (33)	4 (19)	3 (14)	7 (33)
Easier to raise feelings and concerns	11 (52)	2 (10)	2 (10)	6 (29)
Increased shared decision making	6 (29)	5 (24)	5 (24)	5 (24)
Better relationship with professionals	6 (29)	4 (19)	4 (19)	7 (33)

^a^ More than one answer possible.

Ten percent of participants found it beneficial to discuss all PROM responses with a healthcare professional, 33% preferred focusing only on deviant scores, and 57% viewed PROMs as useful solely for quality improvement. The majority (77%) preferred their PROM to be discussed by an obstetric healthcare professional such as Fetal Medicine Specialist, (clinical) midwife, Fetal Medicine doctor (junior doctors with a specialization in ultrasound and fetal medicine), specialized obstetric nurse, medical social worker, or clinical geneticist.

Thirty‐three percent of participants felt PROMs helped prepare for consultations, 38% felt positive about discussing PROM scores with healthcare professionals, and 29% believed PROMs aided in shared decision‐making and advocating for their care. The same percentage (29%) felt PROMs improved their relationship with healthcare professionals. Over half (52%) noted that PROMs helped to raise feelings and concerns during consultations. Analysis of open‐ended responses showed that participants who were neutral or negative about PROMs often had minimal discussion of their PROMs. They cited factors such as limited effort by healthcare professionals to discuss PROM scores, short consultation times, only brief mentions of scores during consultations, and lack of a dedicated healthcare professional as reducing the value of PROMs. Some participants noted an added benefit of gaining personal insight into their thoughts and feelings.

### 3.3 Healthcare Professional Perspectives on the Feasibility of Adapted Setting‐Specific PROMs

Of the 11 healthcare professionals, 82% (*n* = 9) completed the feasibility survey. On average, it was reported that PROMs were discussed for 3 min during consultations (range 0–6 min). Seventy‐five percent of healthcare professionals believed that PROM answers should always be discussed and knew what to address, but only 37.5% knew how to address deviations from normal scores (Table 4). Thematic exploration of open‐ended answers indicated a preference for a clear care pathway and protocol for managing deviated PROM answers. Professionals favored receiving PROM answers before consultations and desired an IT tool for orderly presentation in medical records. The majority felt that the primary treating physician or the midwife in case of shared care pathways should be responsible for discussing PROMs and recommended allocating additional time for these discussions.

**TABLE 4:** Healthcare professional perspectives regarding the use of PROMs in Fetal Medicine, total *N* = 8.

Time investment (minutes), mean (min‐max)	
(Clinical) midwives	2 (0–5)			
Obstetric doctors	0 (0–0)			
Social care worker	2.5 (1–5)			
Overall average	3 (0–3)			
Discuss answers (minutes) mean (min‐max)				
*Mean [min‐max], minutes*				
Clinical midwives	3 (0–6)			
Obstetric doctors	2.5 (0–5)			
Social care worker	2.3 (1–5)			
Overall average	4.5 (3–6)			

**Statements on discussing PROMs, *n* (%)**	**Agree**	**Neutral**	**Disagree**	**Not Applicable**
Knew what to discuss	6 (75)	1 (12.5)	1 (12.5)	0 (0)
Recognized deviants scored answers	7 (87.5)	0 (0)	0 (0)	1 (12.5)
Knew what to do if deviant scores	3 (37.5)	3 (37.5)	1 (12.5)	1 (12.5)
Felt responsible	4 (50)	1 (12.5)	3 (37.5)	0 (0)
Felt that answers should always be discussed	6 (75)	0 (0)	2 (25)	0 (0)
Goal clear	4 (50)	1 (12.5)	0 (0)	3 (37.5)
Capable of explaining purpose	4 (50)	1 (12.5)	0 (0)	3 (37.5)
Easily usable	3 (37.5)	1 (12.5)	1 (12.5)	3 (37.5)
Supports signaling symptoms	4 (50)	2 (25)	0 (0)	2 (25)
Supports identifying what matters to the patient	3 (37.5)	2 (25)	0 (0)	3 (37.5)
Supports appropriate/individualized care	3 (37.5)	3 (37.5)	0 (0)	2 (25)
Supports relationship with patients	3 (37.5)	3 (37.5)	0 (0)	2 (25)
Supports patient empowerment	3 (37.5)	3 (37.5)	0 (0)	2 (25)
Needs insight in answers before consultation	8 (100)	0 (0)	0 (0)	0 (0)
Needs answers directly accessible in EPD	6 (75)	1 (12.5)	1 (12.5)	0 (0)
Needs visual aids (graphs) for answers	3 (37.5)	2 (25)	3 (37.5)	0 (0)

## 4 Discussion

This study evaluated a core set of generic PROMs in Fetal Medicine and identified those relevant for parents facing a fetal anomaly diagnosis: ‘ability to participate in social roles' and ‘emotional distress (anxiety and depression)’ to be used in this specific situation. The pilot study found elevated anxiety but normal depression and social participation scores. Participants found PROMs clear, manageable, and useful for communication, though their value depended on professionals discussing them effectively. Healthcare providers supported using PROMs but sought clearer guidance on handling deviations and preferred an IT tool for better integration into medical records.

The consensus focus group study found unanimously among all groups of stakeholders that only two key PROMs from the generic set were considered relevant for parents during the index pregnancy. This underscores the complexity of Fetal Medicine, where parents act on behalf of a fetus with a congenital anomaly, receiving care and interacting with the Fetal Medicine specialist. A prenatal diagnosis provides information about the health status of the fetus, which can vary greatly, allowing parents to make informed and autonomous reproductive choices. This complexity—where parents' reproductive decisions are influenced by the fetus's condition—makes it challenging to identify a domain and disease specific set of PROMs relevant for this group of patients. This complexity was also evident in the pilot feasibility study. Participants recommended incorporating open‐ended questions or a textbox to enhance personalization in multiple‐choice formats. This aligns with prior research on patient engagement with standardized PROMs in primary and mental health care, highlighting their limitations in capturing the complexity and dynamics of patients' symptoms [16, 17, 18, 19, 20]. In palliative and diabetes care settings, clinicians shared similar concerns, noting that certain standardized PROMs' phrasing may alienate and upset certain patients [21, 22]. This may explain why only one‐third of participants in our pilot feasibility study felt discussing their PROMs helped build relationships with healthcare professionals. Similarly, less than half of clinicians saw benefits for patient‐clinician relationships or patient empowerment. Only one‐third believed that current PROMs identified patients' key concerns or guided individualized care. The desire from parents to include ‘parental stress' as a PROM highlights a need beyond the standard measures of ‘emotional distress (anxiety and depression)’, warranting further exploration in future research. Nonetheless, open‐ended responses indicated that most participants viewed PROMs as valuable tools for exploring emotions, supporting decision‐making, and enhancing care in the Fetal Medicine pathway. The most recognized benefit of the PROM in clinical practice was that it allowed parents to readily raise their feelings and concerns with their healthcare professionals. Furthermore, open data showed that several parents did experience the added benefit of gaining consciousness of their own emotions and discussing this with their direct environment. These benefits reflect evidence across all contexts showing that completion of PROMs supports patients to self‐reflect and identify their needs, values, and barriers [23, 24, 25]. These findings suggest a need for developing and validating a domain‐ and disease‐specific PROMs set for Fetal Medicine, specifically tailored for parents within its unique context.

In this study, one‐third of participants reported that their PROMs were either not discussed at all or barely discussed during consultations. Limited time and insufficient investment by healthcare professionals reduced the effectiveness of PROMs, as only about three minutes were spent discussing them in 15‐min consultations. This often prevented a thorough exploration of patients' thoughts and feelings. This could be attributed to the fact that only 37.5% of professionals knew how to address deviant scored PROM answers. Additionally, healthcare professionals often did not discuss PROMs if scores were normal, merely mentioning the score without exploring the parent's thoughts or feelings.

Professionals emphasized the need for a dedicated care pathway, protocol, and guidance on using and discussing PROM scores in clinical practice. This indicates both a practical implication and a limitation of the current pilot—there was no prior formal training or framework for professionals on when and how to discuss PROMs. To successfully implement PROMs in Fetal Medicine, providing essential training for dedicated professionals is therefore important.

Most participants found the PROM questionnaire manageable, preferred completing it online at home, and needed no assistance, likely due to the younger obstetric patient population's familiarity with digital devices [26, 27].

A reported concern that diminished the patient‐clinician relationship was that participants often saw different healthcare professionals in subsequent consultations. These findings are in accordance with the consensus focus group study results in which stakeholders expressed a strong preference for “Index PROMs” to be discussed by a dedicated case‐manager. Previous research supports the importance of consistency and continuity of care, where parents in Fetal Medicine pathways express a desire to work with the same care team members in consecutive visits, prefer a consistent point of contact and most importantly receive consistent information in similar matters of delivery [28, 29]. The constant change of healthcare professionals affecting the value of PROMs and patient‐clinician relationships highlights how crucial patient‐centered care is for parents in Fetal Medicine pathways [28, 29]. Both the literature and our findings illustrate that closed, multiple choice, standardized PROMs discussed by multiple varying care‐professionals are suboptimal in Fetal Medicine [28, 29]. Therefore, an important implication for practice would be to revise the generic PROM set and in addition develop domain‐ and disease‐specific PROMs tailored for caregivers in Fetal Medicine. A dedicated case manager should review and discuss this PROM set with patients during medical consultations.

### 4.1 Strengths and Limitations

To our knowledge, this pilot study is the first to explore the usability and feasibility of a generic core outcome set in Fetal Medicine. Within the given time frame, it successfully engaged parents and healthcare professionals early in the development and implementation of PROMs for Fetal Medicine, offering preliminary insights into their experiences and preferences and thus identifying important implications for practice and improvements necessary for future PROM use. The small sample size limits statistical relevance and generalizability to all pregnant participants with a (suspected) fetal anomaly. Additionally, the lack of severity stratification in pilot PROM calculations may have affected anomaly distribution. Nonetheless, this study lays the groundwork for future large‐scale research to identify Fetal Medicine‐specific PROMs and enhance generalizability.

## 5 Conclusion

In conclusion, this study provides a first exploration of the development, use and feasibility of PROMS in Fetal Medicine from the perspectives of both parents and professionals. Recommendations for clinical implementation include ensuring continuity of care with a dedicated case manager for personalized PROM discussions and training healthcare professionals on effective engagement in these conversations. Additionally, an accessible IT tool for healthcare professionals to view and discuss PROMs directly in medical records is essential. Finally, there is a need for a tailored set of PROMs specifically designed for caregivers within the unique context of Fetal Medicine.

## Funding

This study was funded by the Netherlands Organisation for Health Research and Development (ZonMw grant no.: 05160472210001).

## Ethics Statement

The Medical Ethical Committee of the UMCU reviewed the protocol (no. 22U‐0426). The Committee declared that the Medical Research Involving Human Subject Act (WMO) did not apply to the present study and therefore exempted the protocol from needing further approval.

## Consent

All participants provided written informed consent in accordance with the protocol and patient information sheet approved by the Medical Ethical Committee.

## Conflicts of Interest

The authors declare no conflicts of interest.

## Supporting information


Supporting Information S1

Supporting Information S2

Supporting Information S3

Supporting Information S4

## Data Availability

The data that support the findings of this study are available on request from the corresponding author. The data are not publicly available due to privacy or ethical restrictions.
