# Supplementary material for: Patient‐Reported Outcome Measures in Fetal Medicine: A Pilot Feasibility Study
Source: Prenat Diagn. 2025 Nov 4;45(13):1757–66. doi: 10.1002/pd.70013 (PMC12693008; doi:10.1002/pd.70013)
Supplement: Supplementary file 1 — Supporting Information S1 [file PD-45-1757-s003.pdf]

Conversation guide

Setup:

Three groups:

- Parents from a previous pregnancy involving a congenital condition in the unborn child, where the decision was made to continue the pregnancy.
- Parents from a previous pregnancy involving a congenital condition in the unborn child, where the decision was made to terminate the pregnancy.
- Healthcare providers involved in pregnancies where a congenital condition is present in the unborn child.

Each group will participate in a physical consensus meeting. If sufficient consensus is not reached, an additional meeting will be held if necessary.

A final consensus meeting will include representatives from all three groups.

- Facilitator:
- Moderator:
- Note-taker/digital support:
- Participants:
- Requirements:
  - Audio recorder
  - Internet access/Miro: https://miro.com/app/board/uXjVPzkROKE=/?share_link_id=175463438218

For parents:

  - ICF form as back-up
  - Parking vouchers
  - Travel cost form

Script for Consensus Meeting:

A. General Start: Introduction and Instructions

1. Welcome (introduction of facilitator and note-taker)
2. Discuss recording, anonymous processing, and purpose (ensure comprehensive notes are taken)
3. Brief introduction of the study and its objectives
4. Introduction round of participants
5. Presentation (PowerPoint) explaining PROs/PROMs

Part 1:

- What is Shared Decision-Making?
- Patient-Reported Outcomes (PROs)
- Generic core set of PROMs
- Use of PROs/PROMs in discussions between healthcare providers and patients

[Slides 1-4]

Part 2:

- The generic core set and the fetal medicine care pathway
- PROs in the care pathway (components of the generic core set)
- The fetal medicine care pathway
- Integrating PROs into the care pathway
- Supporting PROs: for whom, in what way, at which moments, and by whom

Slides [5-6]

B. Start 'Interactive' Session

Materials:

- Miro board with the care pathway

How to:
Participants collaboratively place PROs cards onto the care pathway (indicating when) and discuss whom they relate to. They then assign the appropriate label (who: using categories such as child, family, parent on the respective bars).

[Slides 7-11]

For each PRO/timepoint, the reasoning behind the choice is discussed. Any discrepancies between participants are also addressed. If possible, an attempt is made to reach consensus; if not, the reasons are clearly noted and documented.

Once agreement on the PROs is reached, the discussion focuses on:

- How the PROs will be presented
- By whom the PROs are discussed: fetal specialist, perinatologist/gynecologist, neonatologist, clinical geneticist, medical social worker, pediatric subspecialist (such as nephrologist, neurologist, cardiologist), nurse, others.

What PROs are we still missing?
Prompting with examples from:

ICHOM (REF: Depla AL, Ernst-Smelt HE, Poels M, Crombag NM, Franx A, Bekker MN. A feasibility study of implementing a patient-centered outcome set for pregnancy and childbirth. Health Sci Rep. 2020 Jun 26;3(3):e168. doi: 10.1002/hsr2.168. PMID: 32607452; PMCID: PMC7317300)

- Confidence in the role as a mother
- Mother-child bonding
- Depressive symptoms
- Support from surroundings
- Quality of life

Congenital Diaphragmatic Hernia (REF: Taillieu A, Deprest J, Vergote S, Debeer A, Russo FM, Power B, Meijer F, Crombag N. Patient-reported outcomes for congenital diaphragmatic hernia: A qualitative study. Prenat Diagn. 2023 Mar;43(3):339-354. doi: 10.1002/pd.6303. Epub 2023 Jan 21. PMID: 36598028)

- Survival chances

- Fetal/maternal complications
- Impact on the family
- Mother-child bonding
- Mental well-being of the parents
- Satisfaction with care

What might you feel is missing in the care pathway?

Closing and Thanks

Slides (in Dutch):

A. General Start: Introduction and Instructions

Part 1

Slide 1-4

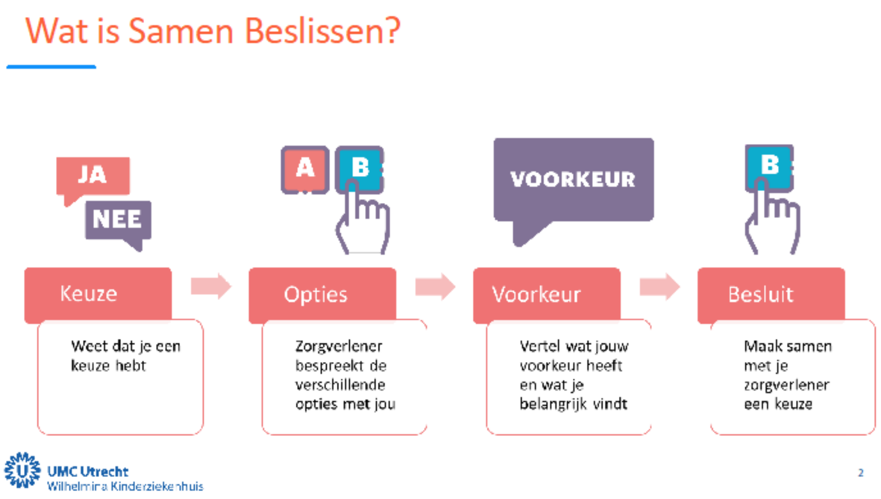

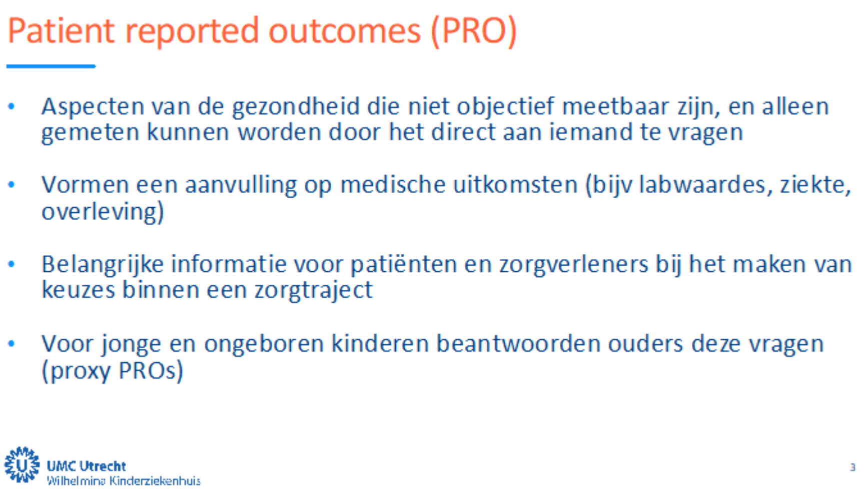

Generieke kernset PRO(M)S

Samenstelling kernset PROs

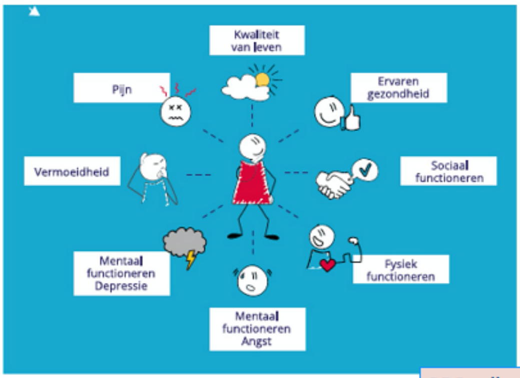

Overkoepelend	• Kwaliteit van leven		
	• Ervaren gezondheid		
Functioneren	• Fysiek functioneren		
	• Sociaal functioneren/ participatie		
	• Mentaal functioneren	Angst	
		Depressie	
Symptomen	• Vermoeidheid		
	• Pijn		

PROs zijn de uitkomsten van zorg
PROMs is het instrument dat deze PROs meet bijv dmv vragenlijst of dagboek

UMC Utrecht
Wilhelmina Kinderziekenhuis

Jouw wensen en verwachtingen

Hoe bereid jij je voor op het gesprek met de zorgverlener?

Wat vertel je de zorgverlener?

Wie maakt uiteindelijk de beslissing?

Wil je naasten hierbij betrekken?

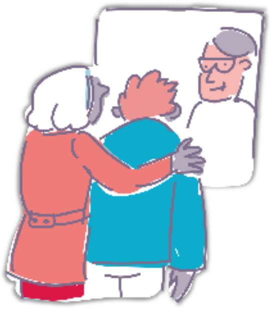

UMC Utrecht
Wilhelmina Kinderziekenhuis

Part 2

Slides 5-6

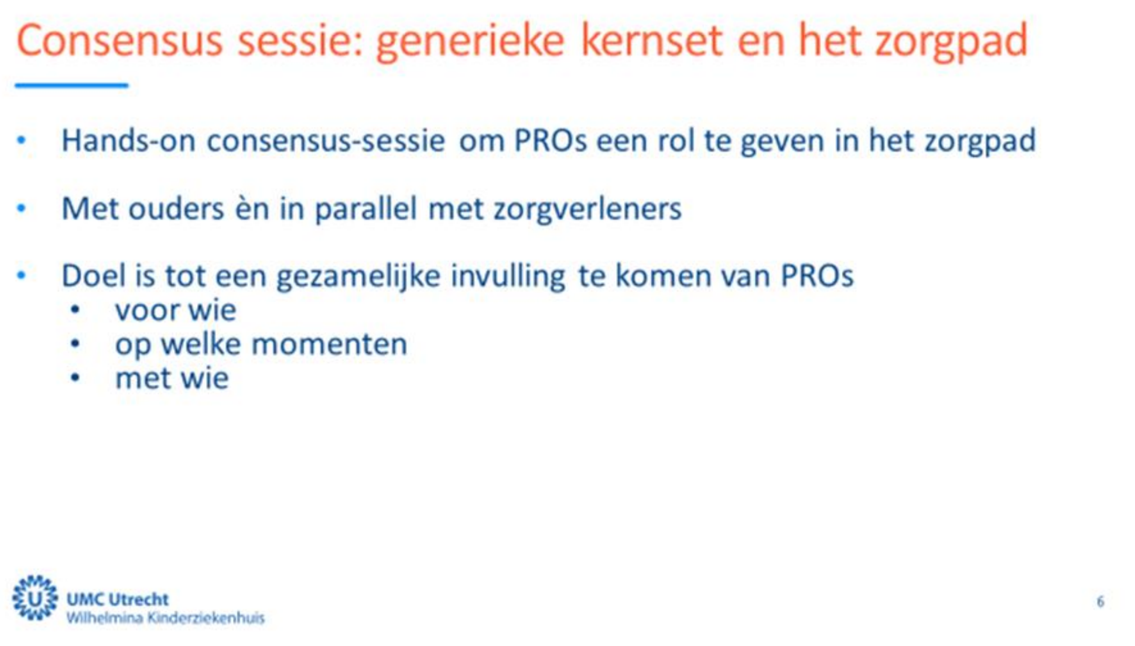

6

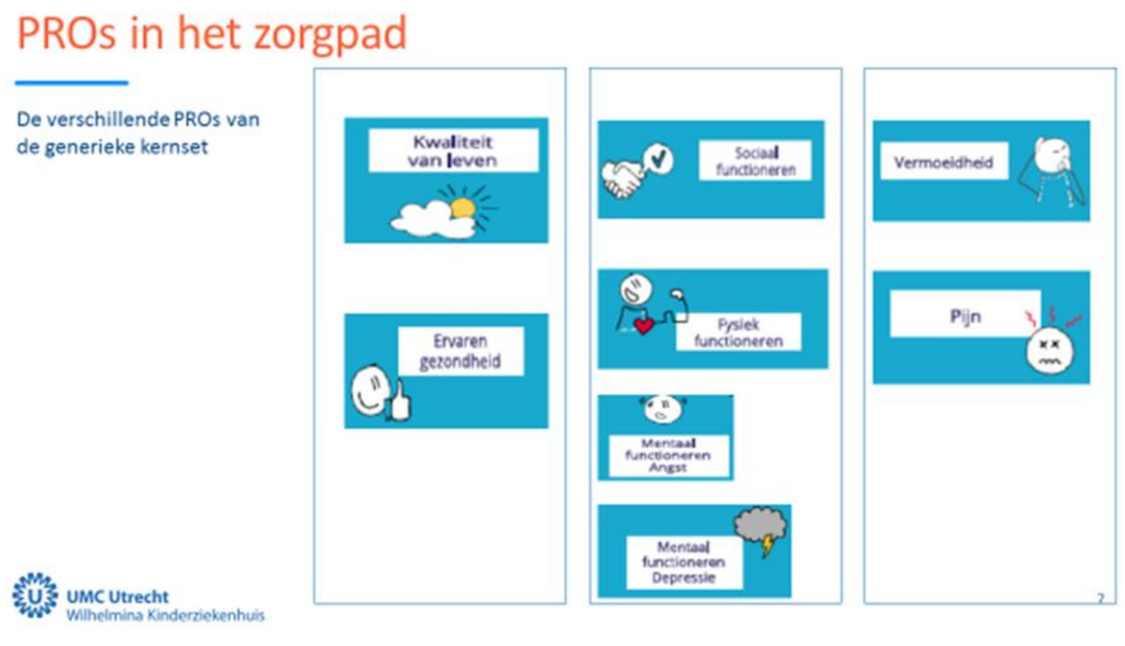

B. Start 'Interactive' Session

Slides 7-11

Zorgpad foetale geneeskunde

Zorgpad

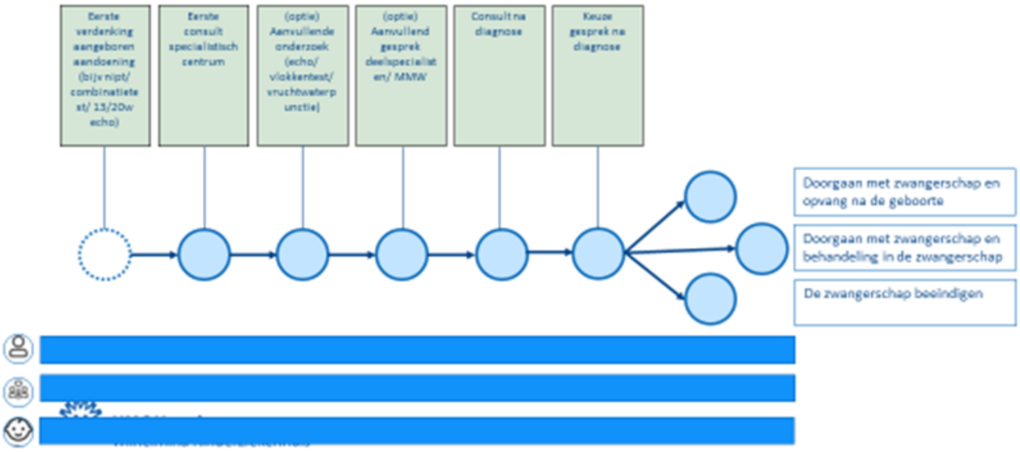

PROs in het zorgpad plaatsen

a) **op wie betrekking**

   a) → *zelf*

   b) → *gezin*

   c) → *het kind* → *Hierbij gaat het over de perceptie van de ouders op (evt) toekomstig leven*

b) **op welke momenten**

c) **met wie: foetale-gynaecoloog, perinatoloog/ gynaecoloog, neonatoloog, klinisch geneticus, medisch maatschappelijk werker, kinder-deelspecialist (zoals bijvoorbeeld nefroloog, neuroloog, cardioloog), verpleegkundige, overige**

PROs op wie betrekking

a) Wat is belangrijk in *mijn* leven
b) Wat is belangrijk in *ons* leven (het gezin)
c) Wat vind ik/wij belangrijk voor het leven van *ons/een kind*

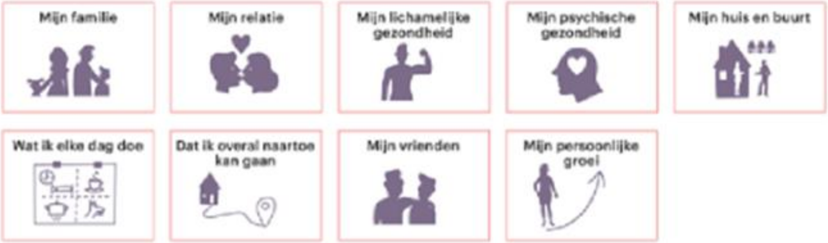

Vorm/ manier

De standaard:

- **Vragenlijst**
- **27 items kernset**
- **Wat is meest passende vorm?**

PROMIS® Item Bank v1.0 – Anxiety – Short Form 4a

Angst

Geef een reactie op elke vraag of uitspraak door per rij één hokje aan te kruisen.

Geef a.u.b. antwoord voor de afgelopen 7 dagen.	Nooit	Zelden	Soms	Vaak	Altijd
Ik voelde me angstig.	☐ 1	☐ 2	☐ 3	☐ 4	☐ 5
Ik vond het moeilijk om me op iets anders dan mijn angst en bezorgdheid te concentreren.	☐ 1	☐ 2	☐ 3	☐ 4	☐ 5
Mijn zorgen waren me te veel.	☐ 1	☐ 2	☐ 3	☐ 4	☐ 5
Ik voelde me slecht op mijn gemak.	☐ 1	☐ 2	☐ 3	☐ 4	☐ 5

	Nooit	Zelden	Soms	Meestal	Altijd
Ik heb moeite om al mijn gewone vrijetijdsactiviteiten met anderen te doen.	☐ 5	☐ 4	☐ 3	☐ 2	☐ 1
Ik heb moeite om alle gezins-/familieactiviteiten te doen die ik wil doen.	☐ 5	☐ 4	☐ 3	☐ 2	☐ 1
Ik heb moeite om al mijn gewone werk (inclusief werk thuis) te doen.	☐ 5	☐ 4	☐ 3	☐ 2	☐ 1
Ik heb moeite om alle activiteiten met vrienden te doen die ik wil doen.	☐ 5	☐ 4	☐ 3	☐ 2	☐ 1

Door wie PROs bespreken?

- Perinatoloog
- Perinatoloog met foetaal aandachtsgebied
- Deelspecialist (neonatoloog, cardioloog, klinisch geneticus, uroloog, neuroloog etc)
- Verpleegkundige
- Echoscopist
- Medisch maatschappelijk werk
- (eerstelijns) verloskundige
- ….?
