# Supplementary material for: Patient‐Reported Outcome Measures in Fetal Medicine: A Pilot Feasibility Study
Source: Prenat Diagn. 2025 Nov 4;45(13):1757–66. doi: 10.1002/pd.70013 (PMC12693008; doi:10.1002/pd.70013)
Supplement: Supplementary file 3 — Supporting Information S3 [file PD-45-1757-s002.pdf]

PROMIS questionnaires

PROMIS Item Bank v1.0-Emotional Distress-Anxiety – Short Form 4a

Emotional Distress-Anxiety – Short Form 4a

Please respond to each question or statement by marking one box per row.
In the past 7 days…

	Never	Rarely	Sometimes	Often	Always
I felt fearful					
I found it hard to focus on anything other than my anxiety.					
My worries overwhelmed me					
I felt uneasy					
	1	2	3	4	5

PROMIS® Item Bank v1.0 – Emotional Distress-Depression – Short Form 4a

Emotional Distress-Depression – Short Form 4a

Please respond to each question or statement by marking one box per row.

In the past 7 days...

	Never	Rarely	Sometimes	Often	Always
I felt worthless					
I felt helpless					
I felt depressed					
I felt hopeless					
	1	2	3	4	5

PROMIS® Item Bank v2.0 - Ability to Participate in Social Roles and Activities- Short Form 4a

Ability to Participate in Social Roles and Activities - Short Form 4a

Please respond to each item by marking one box per row.

	Never	Rarely	Sometimes	Often	Always
I have trouble doing all of my regular leisure activities with others					
I have trouble doing all of the family activities that I want to do					
I have trouble doing all of my usual work (include work at home)					
I have trouble doing all of the activities with friends that I want to do					
	5	4	3	2	1
