# Supplementary material for: Patient‐Reported Outcome Measures in Fetal Medicine: A Pilot Feasibility Study
Source: Prenat Diagn. 2025 Nov 4;45(13):1757–66. doi: 10.1002/pd.70013 (PMC12693008; doi:10.1002/pd.70013)
Supplement: Supplementary file 4 — Supporting Information S4 [file PD-45-1757-s004.pdf]

***Supplementary IV** Fetal and obstetric characteristics of participants (n=27) included in the pilot feasibility study.*

Fetal anomaly	Outcome of pregnancy*
Included during phase I**	
Increased Nuchal Fold	Additional invasive/genetic tests normal, continuation & return to community midwife
NIPT additional finding T3 + T7	Additional invasive/genetic tests normal, continuation of pregnancy
NIPT additional finding T21	Additional invasive/genetic tests normal, continuation of pregnancy
NIPT additional finding T1	Additional invasive/genetic tests normal, continuation of pregnancy
NIPT: suspected for gain 15Q	Additional invasive/genetic tests confirmed, termination of Pregnancy
TGA + Duplex kidney	Continuation of pregnancy
NIPT: suspected for T13	Additional invasive/genetic tests normal, continuation of pregnancy
Included during phase II***	
TGA +VSD	Continuation of pregnancy
CCAM	Continuation of pregnancy
Isolated CCA	Additional invasive/genetic tests normal, continuation & return to community midwife
Mosaicism T16 (45%),<br>Right Aortic arch, persistent left VCS,<br>Splenic cyst	Continuation of pregnancy
T21	Additional invasive/genetic tests T21 confirmed, termination of pregnancy
CCAM	Continuation of pregnancy
CPM T20	Additional invasive/genetic tests normal, continuation of pregnancy
Isolated CCA	Additional invasive/genetic tests normal, continuation of pregnancy
Suspected CPAM	Microcystic pulmonary sequestration, continuation of pregnancy
Hyperechogenic bowel	Additional invasive/genetic tests normal, continuation of pregnancy
CPM T21	Additional invasive/genetic tests normal, continuation of pregnancy
DORV & TGA	TOF, continuation of pregnancy
CPM T8	Additional invasive/genetic tests normal, continuation of pregnancy
Mild hydronefrosis	Continuation of pregnancy
Muscular VSD	Continuation of pregnancy
CPM T13	Additional invasive/genetic tests normal, continuation of pregnancy
T18	Additional invasive/genetic tests confirmed T18, continuation of pregnancy, comfort care
Fetal intra-abdominal cyst	Continuation of pregnancy
Bilateral clubfoot & fetal wrist position anomalies	Continuation of pregnancy
TGA	Continuation of pregnancy

CPM= confined placental mosaicism, CCA = corpus callosum agenesis, CCAM = congenital cystic adenomatoid malformation, CPAM= congenital pulmonary airway malformation, DORV= double outlet right ventricle, NIPT = non-invasive prenatal test, TGA = transposition of the great arteries, T1= trisomy 1, T8 = trisomy 8, T13 = trisomy 13, T18, = trisomy 18, T21= trisomy 21, T20= trisomy 20, VSD = ventricular septum defect.
** All continued pregnancies resulted in live births at full term*
*** Phase I, from suspected to confirmed diagnosis*

**** Phase II, from confirmed diagnosis to pregnancy management (continuation or termination)*
